# Auxin guides roots to avoid obstacles during gravitropic growth

**DOI:** 10.1111/nph.16203

**Published:** 2019-10-11

**Authors:** Yuzhou Zhang, Jiří Friml

**Affiliations:** ^1^ Institute of Science and Technology (IST) Austria 3400 Klosterneuburg Austria

**Keywords:** auxin, gravitropism, obstacle avoidance, PIN auxin transporter, plant evolution, thigmotropism

## Abstract

This article is a Commentary on https://doi.org/10.1111/nph.16076.

Unlike animals, plants are sessile organisms that are unable to flee from adverse conditions. Plants anchor themselves in the soil and must be able to rapidly adapt to an unfavorable or changing environment. Over the course of evolutionary time, in order to colonize diverse habitats on land, plants have evolved formidable capabilities to sense changes in their surroundings and to quickly respond by altering their direction of growth (Sato *et al.*, 2015). These movements, by which plants grow either towards or away from stimuli, such as light and gravity, are termed tropisms. The phenomenon of plant tropism has fascinated botanists for hundreds of years, and this interest led to the identification of the plant hormone auxin and its crucial role in mediating this biological process. The mechanism of root gravitropism evolved in higher plants (Zhang *et al.*, 2019a) and it is driven by the auxin redistribution between the lower and upper side of the root, which is mediated by PIN auxin efflux transporters (Luschnig *et al.*, 1998; Friml *et al.*, 2002; Baster *et al.*, 2013). This auxin gradient leads to differential growth rates that then cause the root to bend in the direction of the Earth's gravity. Similar to gravitropism, root thigmotropism is a crucial process allowing plants to deal with impenetrable obstacles in their surroundings, such as rocks in the soil, facilitating the rapid bending of roots away from the obstacles and finding their edge (Braam, 2005). The combination of both root obstacle avoidance and root gravitropism enables the roots to grow downward into deep soil to access water and nutrients. However, distinct from root gravitropism, in which bending relies on the PIN‐mediated intercellular auxin transport, the mechanism underlying root bending during obstacle avoidance has been largely unknown. In this issue of *New Phytologist*, Lee *et al.* (2020; pp. 1285–1296) unambiguously shows that the first bending of thigmotropism is not simply a passive result of the root growth against a barrier, but it involves active signal transduction that requires asymmetric auxin distribution and differential cell elongation between the convex and concave sides of the *Arabidopsis* root (Lee *et al.*). Additionally, the Lee *et al.* study reveals that besides the PIN‐mediated auxin transport, root obstacle avoidance depends also on the TRANSPORT INHIBITOR RESPONSE1/AUXIN SIGNALING F BOX PROTEIN (TIR1/AFB) auxin signaling pathway. These findings provide crucial insights into the working machinery of root obstacle avoidance.

To better understand the mechanism of obstacle avoidance during root–obstacle interactions, let us compare root gravitropism with root thigmotropism. Generally, root tropism can be divided into three temporally distinct phases: (1) perception of the environmental cue, (2) transmission of the perceived signal and, (3) ultimately, the growth response itself (Sato *et al.*, 2015).

*‘These findings provide crucial insights into the working machinery of root obstacle avoidance.’*

1. For root gravitropism, the root apex is the primary site where gravity is sensed (Sato *et al.*, 2015). The dense starch‐filled amyloplasts within the root apex sediment in the direction of the gravity vector, enabling higher plant roots to perceive the gravity field (Fig. 1). However, it is still unknown which part of the root is used for the obstacle perception, even though it seems plausible that the root apex could be the site of the touch sensor due to its immediate physical interaction with the obstacle. Additionally, it is also unknown which component(s) or organelles in the root cells are utilized to perceive the mechanical force, and how they convert the physical touch signal to the auxin signal.
2. Gravitropic signal transduction involves transmission of auxin signal from the place of gravity perception in the root tip to the place of growth response in the elongation zone and involves both auxin importer and exporter proteins from the AUX1/LAX1 and PIN families, respectively (Sato *et al.*, 2015). Following gravistimulation, the originally symmetrically placed PIN3 and PIN7 are rapidly relocalized to the new bottom side of the columella cells within the root apex, thus diverting auxin flow to the lower side of the root tip (Friml *et al.*, 2002). From there auxin is further transported by the action of PIN2 towards the elongation zone. This asymmetric auxin flow is further reinforced by the PIN2 stabilization in cells along the lower side of the root and its destabilization at the opposite root side (Baster *et al.*, 2013) (Fig. 1). Similar to root gravitropism, asymmetric auxin flow is also observed during root obstacle avoidance, as clearly indicated by the asymmetric expression of auxin response indicators, *DR5rev:GFP* and *DII‐VENUS* (Lee *et al.*). Pharmacological and genetic experiments demonstrate that the auxin asymmetry in thigmotropism also depends on the PIN auxin transporter activity (Lee *et al.*). It remains to be demonstrated, however, whether the PIN relocalization and the asymmetric degradation also occurs during root obstacle avoidance, as it does in root gravitropism (Fig. 1).
3. Once it has arrived at the elongation zone, by the PIN2 action, the auxin signal is ultimately converted to the asymmetric growth, resulting in root bending along the gravity vector. Notably, unlike in root gravitropism, for which the bending initiates in the distal elongation zone (DEZ), the first bending in root thigmotropism occurs in the central elongation zone (CEZ) above the DEZ (Fasano *et al.*, 2002). In gravitropism, auxin inhibits growth at the lower root side by the rapid action of TIR1/AFB auxin signaling (Fendrych *et al.*, 2018). Similarly, root bending in thigmotropism also depends on the TIR1/AFB‐mediated auxin signaling (Lee *et al.*; Zhang *et al.*, 2019b), but the details of the downstream cellular processes leading to growth regulation are unclear in both types of tropism (Fig. 1).

**Figure 1:**
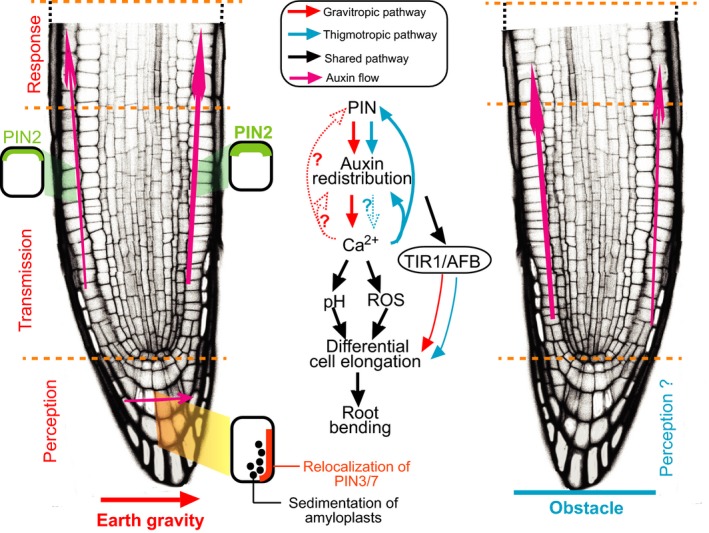
Mechanistic comparison of gravitropism and thigmotropism in roots of higher plants. After horizontal gravistimulation, the sedimentation of amyloplasts within root apex cells triggers both the re‐localization of PIN3/PIN7 within root columella cells and the asymmetric degradation of PIN2 between the upper and lower sides of the root to redirect the intercellular auxin flow along the lower side towards the elongation zone, where auxin inhibits growth. This auxin redistribution coincides with the cytosolic calcium ion (Ca^2+^) waves, which in turn trigger the production of apoplastic reactive oxygen species (ROS) and pH changes that may be linked to growth regulation. During root obstacle avoidance, it is unknown which part of the root is used for touch perception and which component(s)/organelles trigger the PIN‐mediated asymmetric auxin distribution. Ca^2+^ waves were observed in root thigmotropism and have been recognized to facilitate root bending through the downstream pH and ROS signals as in root gravitropism. Blocking Ca^2+^ signaling interferes with the PIN‐mediated auxin redistribution during obstacle avoidance, implying the existence of a feedback loop between the Ca^2+^ wave and the auxin distribution. This can occur through demonstrated Ca^2+^ effect on PIN subcellular distribution. Both root gravitropism and thigmotropism rely on the TIR1/AFB‐mediated auxin signaling pathway to regulate differential epidermal cell elongation and ultimately the root bending.

In addition to auxin, several other signals, such as calcium ion (Ca^2+^), pH and reactive oxygen species (ROS) have been implicated in the regulation of both root gravitropism and thigmotropism (Nakagawa *et al.*, 2007) (Fig. 1). During root bending, the cytosolic Ca^2+^ concentration rapidly increases in the epidermal cells on the convex root side, and the increased Ca^2+^ levels are linked with the production of apoplastic ROS and cytoplasmic acidification, which are involved in cell elongation (Monshausen *et al.*, 2009). Regarding root gravitropism, it has been observed that auxin distribution is somehow linked to the cytosolic Ca^2+^ wave (Sato *et al.*, 2015; Su *et al.*, 2017) and Ca^2+^, in turn, regulates PIN localization and gravitropism (Zhang *et al.*, 2011). It seems plausible that the PIN‐mediated auxin redistribution in root thigmotropism may also trigger Ca^2+^ signaling; however, it remains to be addressed. Notably, the Lee *et al.* study also nicely shows that inhibition of Ca^2+^ signaling blocks the asymmetric auxin distribution, suggesting a more complex feedback regulation between the auxin and Ca^2+^ signaling pathways during root bending in both contexts. Among the outstanding questions are whether auxin and Ca^2+^ act in a common or parallel signaling pathway in the regulation of tropisms and what are the upstream and downstream processes?

Notably, observation of *Arabidopsis* root movement has revealed that root bending takes place within 20 min after contact with the barrier (Lee *et al.*), indicating that root obstacle avoidance might be a rapid process. Recently, it has been shown that root gravitropism in flowering plants is also highly efficient when compared with that in basal vascular plants such as lycophytes and ferns (Zhang *et al.*, 2019a). These results imply that, to better adapt to terrestrial environments with constantly changing environments, higher plants evolved more efficient tropism responses compared with their predecessors, to facilitate their successful colonization of the land. Rapid, efficient root gravitropism evolved in seed plants (i.e. gymnosperms and flowering plants); nevertheless, it is unclear when during evolution active, efficient thigmotropism appeared (Fig. 2). Studies have revealed that efficient tropism relies on the PIN3 clade (PIN3/4/7 in *Arabidopsis*) and the PIN2 clade in flowering plants (Zhang *et al.*, 2019a), since these PIN genes are able to rapidly adjust and redirect the auxin flow (Su *et al.*, 2017). Analysis of the PIN phylogeny and comparison of the root structures implies that efficient thigmotropism could have also evolved only in seed plants. Based on the phylogenetic tree of PIN protein sequences, the gymnosperm PINH/G proteins are in the same clade as flowering plant PIN2, and interspecies complementation reveals that their functions are equivalent in root gravitropism, while the gymnosperm PINE groups in the same clade with flowering plant PIN3/4/7 (Fig. 2). However, the proteins in the two PIN clades are not present in lower plants including the ferns and lycophytes (Bennett *et al.*, 2014) (Fig. 2). Additionally, the anatomic root structure of seed plants is quite distinct as compared to the roots of early diverging extant vascular plants, lycophytes and ferns (Zhang *et al.*, 2019a). Thus, it still needs to be clarified whether basal vascular plants possess similar thigmotropic activity as found in flowering plants, and if so, if their roots utilize the PIN proteins from the other clades to regulate this process. Such studies would shed light on an eventual co‐evolution of root thigmotropism and gravitropism, revealing how plants after colonizing land ‘learned’ to deal with challenging conditions of growing through different types of soils.

**Figure 2:**
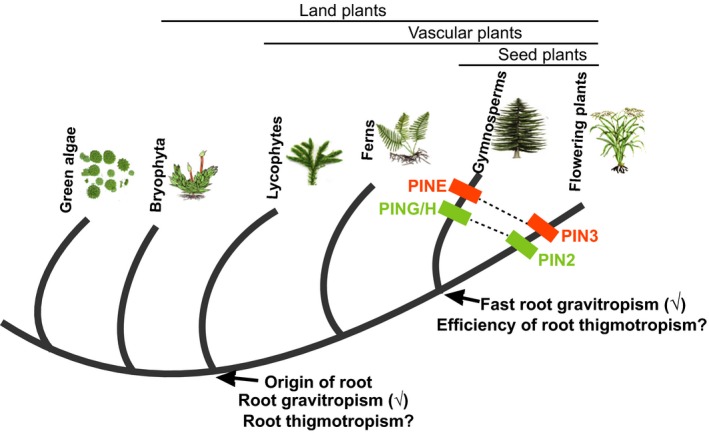
A model for the evolution of root gravitropism and thigmotropism. True roots evolved in the vascular plant lineages; roots of the early diverging vascular plants such as ferns and lycophytes only show slow, rudimentary root gravitropism, but whether they also evolved thigmotropism is still unknown. The fast, effective PIN‐mediated root gravitropism evolved in seed plants, accompanied by the emergence of the PIN clade containing gymnosperm PING/H and angiosperm PIN2 proteins, as well as the gymnosperm PINE and angiosperm PIN3 clade. Although the two PIN clades are also critical for thigmotropism as reported in this issue of *New Phytologist* by Lee *et al.* (2020; pp. 1285–1296), additional evidence is needed to show whether the efficient root thigmotropism only evolved in the seed plants.
